# Evaluation of antiseizure drug efficacy and tolerability in the rat lamotrigine‐resistant amygdala kindling model

**DOI:** 10.1002/epi4.12354

**Published:** 2019-08-12

**Authors:** Cameron S. Metcalf, Jennifer Huff, Kyle E. Thomson, Kristina Johnson, Sharon F. Edwards, Karen S. Wilcox

**Affiliations:** ^1^ Anticonvulsant Drug Development Program, Department of Pharmacology and Toxicology University of Utah Salt Lake City UT USA

**Keywords:** animal models, antiseizure drugs, kindling, pharmacoresistant epilepsy

## Abstract

**Objective:**

The lamotrigine‐resistant amygdala kindling model uses repeated administration of a low dose of lamotrigine during the kindling process to produce resistance to lamotrigine, which also extends to some other antiseizure drugs (ASDs). This model of pharmacoresistant epilepsy has been incorporated into the testing scheme utilized by the Epilepsy Therapy Screening Program (ETSP). Although some ASDs have been evaluated in this model, a comprehensive evaluation of ASD prototypes has not been reported.

**Methods:**

Following depth electrode implantation and recovery, rats were exposed to lamotrigine (5 mg/kg, i.p.) prior to each stimulation during the kindling development process (~3 weeks). A test dose of lamotrigine was used to confirm that fully kindled rats were lamotrigine‐resistant. Efficacy (unambiguous protection against electrically elicited convulsive seizures) was defined as a Racine score < 3 in the absence of overt compound‐induced side effects. Various ASDs, comprising several mechanistic classes, were administered to fully kindled, lamotrigine‐resistant rats. Where possible, multiple doses of each drug were administered in order to obtain median effective dose (ED_50_) values.

**Results:**

Five sodium channel blockers tested (eslicarbazepine, lacosamide, lamotrigine, phenytoin, and rufinamide) were either not efficacious or effective only at doses that were not well‐tolerated in this model. In contrast, compounds targeting either GABA receptors (clobazam, clonazepam, phenobarbital) or GABA‐uptake proteins (tiagabine) produced dose‐dependent efficacy against convulsive seizures. Compounds acting to modulate Ca^2+^ channels show differential activity: Ethosuximide was not effective, whereas gabapentin was moderately efficacious. Ezogabine and valproate were also highly effective, whereas topiramate and levetiracetam were not effective at the doses tested.

**Significance:**

These results strengthen the conclusion that the lamotrigine‐resistant amygdala kindling model demonstrates pharmacoresistance to certain ASDs, including, but not limited to, sodium channel blockers, and supports the utility of the model for helping to identify compounds with potential efficacy against pharmacoresistant seizures.

**Key Points**

- Drug‐resistant kindling models are useful for studying pharmacoresistant epilepsy
- Although the lamotrigine‐resistant amygdala‐kindled rat model has been described previously, to date no comprehensive pharmacology has been reported
- Of those compounds tested, only clonazepam, ezogabine, and valproate were found to be effective in this model
- Tolerability assessments in seizure‐ and drug‐experienced animals may yield notably different results compared with naïve animals

## 1 INTRODUCTION

Despite the availability of more than 20 antiseizure drugs (ASDs), one‐third of patients with epilepsy do not experience adequate seizure control. While acute screening models in mice and rats such as the maximal electroshock and subcutaneous pentylenetetrazole models can help to identify novel ASDs, these models are not able to identify some compounds with antiseizure effects and can lead to false assumptions of efficacy in other compounds.^1^ In this way, additional models can aid in identification and differentiation of novel compounds. Further, in order to find compounds with potential efficacy against drug‐resistant seizures, models should demonstrate that few of the currently available antiseizure drugs are effective in blocking seizures.^1^

For many years, it has been known that lamotrigine administration during kindling acquisition can lead to a subsequent resistance and decrease in drug efficacy.^2^ Further, it was observed that tolerance to lamotrigine extended to some sodium channel blockers but not to ASDs with other mechanisms.^3^ Further, kindled rats can display sensitivity or resistance to other ASDs (eg, phenobarbital and phenytoin),^4^, ^5^, ^6^, ^7^, ^8^, ^9^ and genetic factors or animal strain can influence sensitivity to ASDs (eg, phenytoin) in kindled rats.^7^, ^8^ Further, resistance to one drug can extend to other ASDs,^5^, ^6^, ^10^ thus suggesting the potential for this approach as a screening model for drug‐resistant epilepsy. The lamotrigine‐resistant amygdala kindling model utilizes repeated administration of lamotrigine during the kindling process wherein animals develop a resistance to lamotrigine, which also extends to other some other ASDs.^10^, ^11^ Therefore, this model has been incorporated as a late‐stage differentiation assay in a pharmacoresistance screening workflow utilized by the Epilepsy Therapy Screening Program (ETSP).^12^, ^13^

One of the major goals for the development of novel ASDs is to minimize or eliminate side effects at therapeutic doses.^14^, ^15^ Newer ASDs have provided specific advantages over previously existing therapies, but substantial gains in efficacy and tolerability are still needed.^16^ Further, despite the availability of a variety of animal models to aid in predictions of efficacy, initial tolerability estimates are typically performed in drug‐ and seizure‐naïve young adult animals.^12^ At an early phase of preclinical drug evaluation, this approach is beneficial as it is inexpensive and informative for later studies. However, it is likely that differences in drug responses, including untoward effects, may not be adequately represented by tolerability studies performed in naïve animals. Therefore, the evaluation of tolerability in relevant disease models (ie, in animals with behavioral seizures) may help to elucidate potential adverse behaviors or untoward responses that do not occur in seizure‐ and/or drug‐naïve animals.^17^

Although carbamazepine, valproate, and ezogabine have been evaluated in the lamotrigine‐resistant amygdala‐kindled rat model,^1^, ^10^, ^11^ a comprehensive assessment of ASD prototypes has not been reported. Thus, it is currently unclear whether certain classes of ASDs are effective in this model. Moreover, initial tolerability data obtained in seizure‐ and drug‐naïve young adult rats^18^, ^19^ may not be replicated in seizure‐experienced (eg, fully kindled) rats. In this way, tolerability assessments in kindled rats may be more translatable to clinical epilepsy populations. Therefore, we sought to evaluate several prototype ASDs, comprising several mechanisms of action, in this model. These data will serve as key comparators for novel antiseizure compounds, and help to identify those with activity against pharmacoresistant epilepsy.

## 2 METHODS

### 2.1 Compound preparation

All compounds were prepared in 0.5% methylcellulose (Sigma; St. Louis, MO, USA) suspensions, with the exception of sodium valproate, which was prepared in saline (0.9% NaCl). Carbamazepine, clobazam, clonazepam, ethosuximide, ezogabine, phenobarbital, phenytoin, and sodium valproate (valproate) were obtained from Sigma. Eslicarbazepine, gabapentin, levetiracetam, rufinamide, tiagabine, and topiramate were obtained from TCI America. Lacosamide was obtained from Axon Medchem. Lamotrigine was obtained from AK Scientific.

### 2.2 Animals

Adult male Sprague‐Dawley rats (240‐251 g; Charles River) were group‐housed (4 animals/cage) in a temperature‐ and humidity‐controlled facility and maintained on a constant 12‐hour light/dark cycle with free access to standard rat chow and water. After delivery, animals were allowed sufficient time to acclimate to housing conditions prior to surgery (~1 week). The animals were housed and fed in a manner consistent with the recommendations in the “Guide for Care and Use of Laboratory Animals” (National Research Council). Housing, handling, and testing were performed in accordance with Public Health Service policy guidelines and a protocol approved by the Institutional Animal Care and Use Committee of the University of Utah.

### 2.3 Surgical placement of kindling electrode

Rats were anesthetized with Fluriso (Isoflurane, USP, MWI Animal Health) using a Vetequip (Livermore, CA) anesthesia system under aseptic conditions. Animals then received a single subcutaneous (s.c.) dose of buprenorphine (0.2‐0.5 mg/kg; MWI Animal Health) for pain management. A bipolar electrode (Plastics One) was implanted stereotaxically into the right basolateral amygdala (stereotaxic coordinates with reference to bregma, Paxinos, and Watson: anterio‐posterior ‐ 2.2, mediolateral ‐ 4.7, and dorsoventral ‐ 8.7). Implanted electrodes consisted of two twisted, Teflon‐coated stainless steel wires. The electrode assembly was anchored to the skull by three stainless steel screws and fixed in place using dental acrylic. Following surgery, animals received a single injection of penicillin G (60 000 U, s.c.; MWI Animal Health) and rimadyl (0.03 mg/kg, s.c.; MWI Animal Health) and were allowed to recover for 1 week. Following surgery, animals were housed singly for the remainder of the study.

### 2.4 Establishment of lamotrigine‐resistant amygdala‐kindled rats

Rats received daily administrations of intraperitoneal (i.p.) lamotrigine (5 mg/kg; 0.04 mL/10g body weight) during the kindling acquisition phase. On each day of kindling acquisition (5 days/week, 3–4 weeks), 1 hour following lamotrigine administration, animals were connected via tethered electrode (Plastics One) to a Biopac MP100 system, which allowed for baseline and poststimulation electroencephalographic (EEG) recordings. Following a brief (10 seconds) baseline EEG recording, rats received a subthreshold stimulation (200 μA, 50 Hz, 2 seconds) followed by a 3‐minute behavioral observation and EEG recording period. Seizure severity was assessed using a modified Racine^20^, ^21^ scale (0—no response, 1—mouth and facial movement including jaw clonus, 2—head nodding, 3—unilateral forelimb clonus with slight rearing, 4—rearing with unilateral or bilateral forelimb clonus, and 5—loss of the righting reflex including rearing and falling, with forelimb clonus). Animals were considered fully kindled when they showed four or five consecutive generalized seizures (ie, scores of 4‐5). In addition, following each stimulation, an afterdischarge duration was determined as the initial period of high‐amplitude and high‐frequency activity following each kindling stimulation. Afterdischarges were determined visually by an experienced experimenter. Two days following the kindling acquisition period, all animals received a lamotrigine challenge dose (30 mg/kg, i.p.) to confirm lamotrigine resistance (ie, that seizures persisted despite pretreatment with this dose of lamotrigine). Approximately 31% of rats (data not shown) that complete the kindling paradigm demonstrate sensitivity to lamotrigine^11^, despite daily treatment and kindling stimulation. Only animals that demonstrated lamotrigine resistance were included for the evaluation of prototype ASDs.

### 2.5 Evaluation of prototype ASDs in lamotrigine‐resistant fully kindled rats

Prior to drug testing, a baseline stimulation was administered the day prior in order to verify generalized behavioral seizure activity and obtain a baseline afterdischarge duration. Groups of 6‐8 rats were used for each treatment condition. This group size was determined using power analysis, which suggests that groups of this size are sufficient to detect differences in afterdischarge duration of 30 seconds and differences in group efficacy (protection) of 50%. Test compounds were administered using an optimal fluid volume to body fluid ratio (0.04 mL/10g body weight). All compounds were administered by i.p. injection to fully kindled rats that demonstrated resistance to lamotrigine. A battery of standard ASDs was administered using a time‐to‐peak effect consistent with that identified in either maximal electroshock or 6‐Hz seizure tests in rats (data not shown, ETSP contract site historical time‐to‐peak effect evaluation studies; see also Metcalf et al. 2017^19^). Carbamazepine, clonazepam, ethosuximide, lacosamide, lamotrigine, phenytoin, rufinamide, tiagabine, and valproate were administered 0.25 hours prior to testing. Clobazam, eslicarbazepine, and ezogabine were administered 0.5 hours prior to testing. Levetiracetam was administered 1 hour prior to testing. Gabapentin, phenobarbital, and topiramate were administered 2 hours prior to testing. In order to maximize efficiency for use of this model as a screening test, rats were reused for multiple tests, with a minimum of 3‐7 days between tests and a maximum of 10 tests per rat. Experimenters were blinded to the compound (eg, compound was assigned a number) but were not blinded to treatment dose administration. Randomization was not used to allocate animals into dose treatment groups.

#### 2.5.1 Evaluation of behavioral seizure activity

Following treatment, animals were stimulated using the same stimulus intensity used for daily stimulation during kindling acquisition (described above). Behavioral seizures were scored according to the Racine scale,^20^, ^21^ and animals with behavioral seizure scores of 0‐2 were considered “protected”.^11^, ^22^, ^23^ For each compound evaluated, multiple doses were used in order to obtain a median effective dose (ED_50_), when possible.

#### 2.5.2 Evaluation of afterdischarge duration

Afterdischarge duration was obtained for each animal evaluated by examination of high‐frequency and high‐amplitude activity occurring after amygdala stimulation, as described previously.^24^, ^25^, ^26^ A brief baseline (~10 seconds) EEG period, captured prior to the initiation of amygdala stimulation, was used as a comparator to determine the duration of primary afterdischarge activity. Therefore, termination of primary afterdischarge activity was defined when EEG activity returned to baseline levels for a minimum of 3 seconds. Only primary afterdischarge activity was measured during the 180‐second period following amygdala stimulation.

### 2.6 Evaluation of motor impairment

Motor impairment (minimal motor impairment assay) following ASD administration in fully kindled rats was evaluated in a manner similar to that described previously^19^ for drug‐ and seizure‐naïve young adult rats, where rats were evaluated for the following signs: abnormal gait, abnormal body posture, tremors, hyperactivity, lack of exploratory behavior, somnolence, stupor, catalepsy, changes in muscle tone, or hypoactivity. In order to minimize untoward effects on animals, when unfavorable tolerability was observed for approximately half of the animals in a given treatment group, no additional higher doses were used.

### 2.7 Statistical analysis

Afterdischarge duration data are presented as means ± SE. Behavioral seizure activity is presented as the number of protected animals/number tested in each treatment group. ED_50_ values were obtained using behavioral seizure scores for multiple doses of a given compound and were determined using a Probit analysis.

## 3 RESULTS

**Figure 1:**
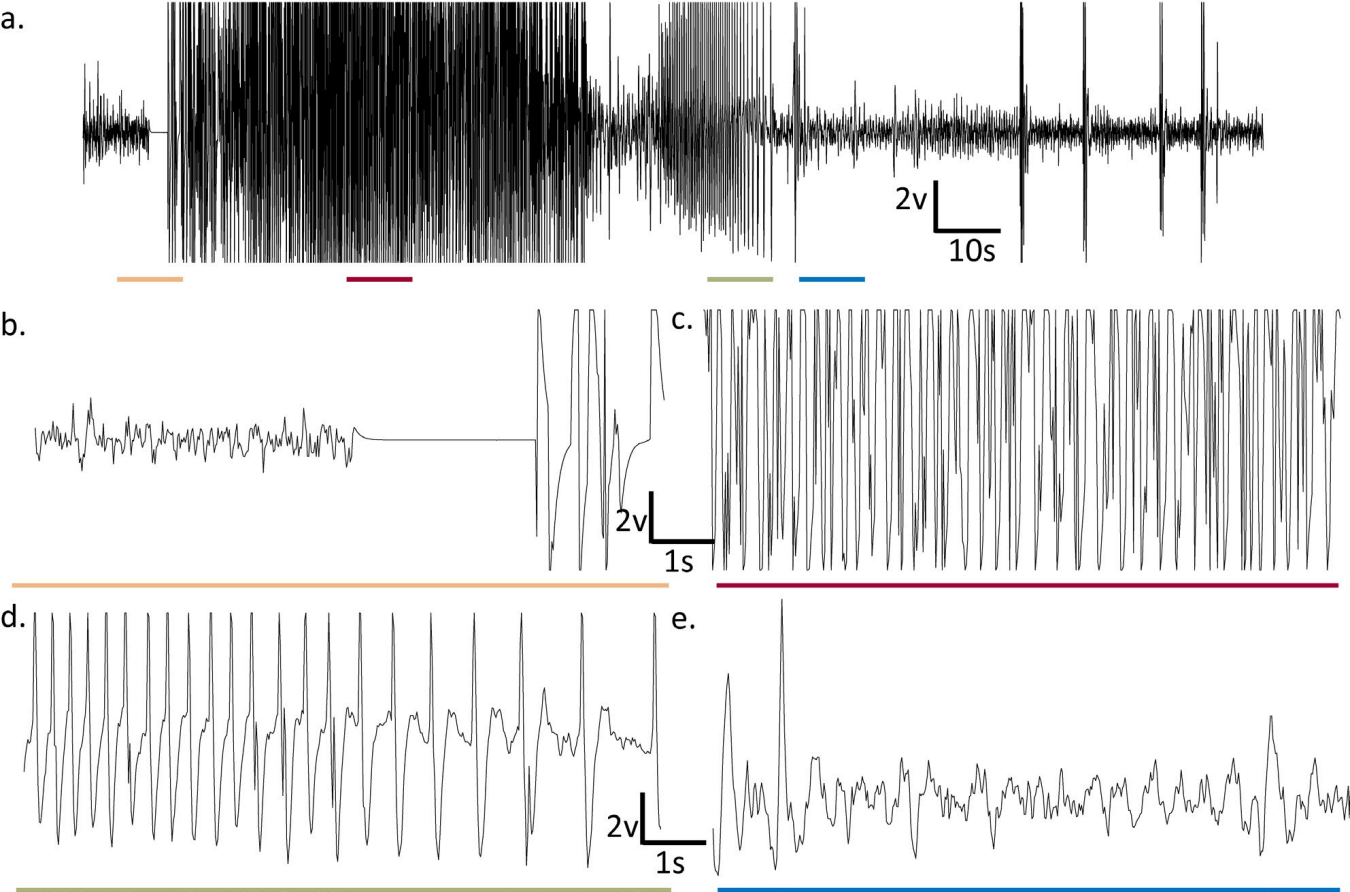
Representative EEG trace prior to and following amygdala stimulation in a lamotrigine‐resistant fully kindled rat. (A) Full EEG trace (180 s) with 10‐s portions underlined for (B‐E). The primary afterdischarge portion is indicated. (B) 10‐s period corresponding to the orange line in (A); baseline recording period with stimulation artifact (3 s). (C) 10‐s period corresponding to the red line in (A); post‐stimulation EEG activity coincident with behavioral seizure activity (racine 4–5 seizures, not shown). (D) 10‐s period corresponding to the green line in (A); reduction in afterdischarge burst frequency corresponding to cessation of behavioral seizure activity (not shown). (E) 10‐s period corresponding to the blue line in (A); approximate return to baseline EEG activity (eg, a minimum of 3 s between bouts of high‐amplitude and high‐frequency activity). Sampling occurred at 50 Hz. The sample trace was obtained from an animal treated with rufinamide (40 mg/kg) and was not protected (ie, generalized behavioral seizure activity was observed)

Prototype ASDs were evaluated at initial doses informed by activity in maximal electroshock or 6‐Hz seizure activity in rats.^19^ Dose levels were increased until untoward effects were observed in at least one animal in the group, at which point no additional (higher) doses were used. Figure 1 depicts a typical afterdischarge following amygdala stimulation in a fully kindled, lamotrigine‐resistant rat. Afterdischarge activity in vehicle‐treated and non‐protected rats generally included a primary afterdischarge of approximately 70‐100 seconds (Figure 1A) followed by additional intermittent high‐frequency and high‐amplitude activity observed through the 3 minutes poststimulation period (see also Figure 1B‐E). Table 1 includes a summary of each compound evaluated in fully kindled lamotrigine‐resistant rats and includes behavioral seizure efficacy, tolerability, and afterdischarge duration for each treatment group. Further, Table 2 includes a summary of the ED_50_ values obtained for behavioral seizure activity for each compound evaluated. In addition, previously published median toxic dose (TD_50_) values for each prototype compound (obtained in young adult, drug‐ and seizure‐naïve animals^19^) are compared to estimated TD_50_ values obtained during testing in lamotrigine‐resistant kindled rats, with the exception of topiramate. For topiramate, a separate study in naïve rats was conducted to obtain a new TD_50_ value.

**Table 1:** Prototype antiseizure drugs evaluated in the lamotrigine‐resistant amygdala kindling model following intraperitoneal administration

Compound	Dose (mg/kg)	Behavioral Seizure Scores	Seizure proportion (# nonconvulsive^a^/total seizures)	Efficacy (# protected^b^/N)	Tolerability (# impaired/N)	Afterdischarge Duration	
						Predrug	Postdrug
Vehicle^c^	0	5,5,5,5,5,5,5	0/8	0/8	0/8	109 ± 17	95 ± 17
Carbamazepine	15	5,0,4,5,5,5,5	0/6	1/7	0/7	89 ± 15	73 ± 14
	30	5,5,2,1,5,5,1,5	3/8	3/8	0/8	125 ± 19	48 ± 16*
	40	0,5,2,0,5,5,2,5	2/6	4/8	0/8	60 ± 8	32 ± 12
	80	0,2,1,2,0,0,3,4	3/5	6/8	6/8	80 ± 21	20 ± 12*
Clobazam	5	5,4,5,5,5,5,2,5	1/8	1/8	0/8	54 ± 5	82 ± 7*
	10	2,2,5,5,5,1,1	4/7	4/7	0/6	89 ± 21	75 ± 6
	15	2,1,1,5,1,2	5/6	5/6	6/6	65 ± 4	50 ± 10
Clonazepam	0.25	5,5,5,5,5,5,5,2	1/8	1/8	0/8	49 ± 9	49 ± 6
	0.5	5,5,5,5,5,1,1	2/7	2/7	0/7	45 ± 5	53 ± 9
	2	2,1,2,2,1,1,1,1	8/8	8/8	0/8	86 ± 15	44 ± 8*
	2.36	5,2,2,5,2,2,2,5	5/8	5/8	0/8	87 ± 18	75 ± 15*
	4	1,2,2,2,2,2,5	6/7	6/7	6/7	58 ± 15	31 ± 6*
Eslicarbazepine	100	4,1,4,5,4,3,4	1/7	2/7	7/7	147 ± 13	68 ± 17*
	150	5,1,5,4,4,4	1/6	1/6	5/6	99 ± 21	74 ± 7
Ethosuximide	200	5,5,5,5,1,5,0,5	1/7	2/8	8/8	107 ± 14	101 ± 18
Ezogabine	1	1,5,5,5,5,5,5,5	1/8	1/8	0/8	107 ± 14	94 ± 18
	5	1,2,0,5,0,3,4	2/5	4/7	0/8	72 ± 11	20 ± 10*
	10	0,0,0,0,1,0,1	2/2	7/7	0/7	85 ± 15	11 ± 3*
	23	0,1,0,0,0,0,0	1/1	7/7	7/7	111 ± 19	8 ± 2*
Gabapentin	10	5,5,5,5,5,5,5	0/7	0/7	0/7	90 ± 21	92 ± 14
	35	2,4,5,4,3,4,3	1/7	1/7	0/7	84 ± 15	64 ± 6
	75	5,2,3,5,4,4,3	1/7	1/7	0/7	112 ± 17	73 ± 10
	150	1,5,4,4,3,3,3	1/7	1/7	0/7	73 ± 10	54 ± 8
	300	2,2,2,2,3,3,2	5/7	5/7	0/7	54 ± 8	17 ± 7*
Lacosamide	10	5,5,5,5,2,5	1/6	1/6	0/6	89 ± 14	62 ± 11
	15	5,5,5,5,4,0,4	0/6	1/7	0/7	99 ± 21	77 ± 19
	30	5,5,3,5,5,4,4	0/7	0/7	0/7	107 ± 21	58 ± 6*
	60	3,0,3,3,3,3,3	0/6	1/7	7/7	58 ± 7	44 ± 10
Lamotrigine	10	5,5,5,0,5,5,5	0/6	1/7	0/7	55 ± 16	47 ± 10
	20	5,5,5,5,5,5,5,5	0/8	0/8	0/8	55 ± 8	63 ± 17
	30	5,5,5,5,5,5,5,5	0/8	0/8	0/8	134 ± 17	109 ± 16
	40	2,5,2,2,3,5,3,4	3/8	3/8	0/8	70 ± 14	94 ± 22
	50	3,5,5,5,5,2,5	1/7	1/7	7/7	106 ± 16	130 ± 18
Levetiracetam	50	4,5,2,5,2,5,5,5	2/8	2/8	0/8	80 ± 16	96 ± 11
	100	3,5,4,4,2,5,1	2/7	2/7	0/7	67 ± 13	80 ± 15
	200	5,5,2,5,2,5,5	2/7	2/7	0/7	85 ± 9	95 ± 17
	400	2,4,5,5,2,2,4	3/7	3/7	0/7	70 ± 14	71 ± 26
Phenobarbital	15	1,4,4,4,5,3,4,5	2/8	1/8	0/7	78 ± 10	76 ± 16
	30	2,5,1,1,1,4,1	5/7	5/7	0/7	86 ± 12	52 ± 16*
	45	1,1,2,1,0,0,1	5/5	7/7	7/7	92 ± 13	39 ± 9*
Phenytoin	10	5,5,5,5,5,5,5,5	0/8	0/8	0/8	68 ± 10	62 ± 14
	20	5,5,5,5,5,5,5,5	0/8	0/8	0/8	55 ± 9	69 ± 12
	30	5,5,5,5,5,5,5	0/7	0/7	0/7	97 ± 19	100 ± 12
	40	4,4,5,4,4,5,4,4	0/8	0/8	2/8	101 ± 20	98 ± 6
Rufinamide	40	4,5,5,5,4,5,5,5	0/8	0/8	8/8	47 ± 10	60 ± 7
Tiagabine	2.5	5,5,5,5,5,5,5	0/7	0/7	0/7	82 ± 13	69 ± 7
	3.75	5,5,5,5,5,5,5	0/7	0/7	0/7	96 ± 19	87 ± 15
	4.5	5,5,5,5,1	1/5	1/5	0/5	60 ± 5	78 ± 17
	5	5,2,2,3,5,2,5	3/7	3/7	0/7	59 ± 12	42 ± 17
	10	0,2,1,3,0,0,1,0	3/4	7/8	8/8	70 ± 9	29 ± 14*
Topiramate	300	5,5,5,5,1,4,4	1/7	1/7	5/7	105 ± 13	89 ± 19
Valproate	75	5,5,5,5,5,5,5,5	0/8	0/8	0/8	49 ± 5	60 ± 6
	135	0,5,0,1,0,2,0,5	2/4	6/8	0/8	66 ± 8	77 ± 19*
	200	0,2,0,0,0,0,0,5	1/2	7/8	0/8	58 ± 5	54 ± 19*
	300	0,0,0,0,0,0,0,0	0/0	8/8	0/8	67 ± 15	20 ± 13*

a Nonconvulsive seizures: Racine scores of 1–2.

b Definition of protected: Racine scores of 0–2.

c 0.5% methylcellulose.

* *P* < 0.05

**Table 2:** Prototype antiseizure drugs evaluated for efficacy in the lamotrigine‐resistant amygdala kindling model and motor impairment (minimal motor impairment assay) following intraperitoneal administration

Compound	Lamotrigine‐resistant Amygdala Kindling Model	Minimal Motor Impairment	
	ED_50_ (mg/kg) (95% confidence interval)	TD_50_ (95% confidence interval)	
		Naïve rats	Kindled rats (estimated)
Carbamazepine	42.1 (21.8–121)	36.7 (21.7–45.1)^a^	~60
Clobazam	9.2 (5.7–14.9)	15.7 (8.0–24.3)^a^	10–15
Clonazepam	0.9 (0.4–1.6)	0.5 (0.3–1.9)^a^	2.4–4
Eslicarbazepine	>150	>100^a^	<100
Ethosuximide	>200	189 (140–228)^a^	<200
Ezogabine	3.2 (1.3–5.4)	42.1 (37.7–48.1)^a^	10–23
Gabapentin	268^b^	156 (142–176)^a^	>300
Lacosamide	>60	13.0 (7.9–19.9)^a^	~45
Lamotrigine	>50	>50^a^	40–50
Levetiracetam	>400	>100^a^	>100
Phenobarbital	23.3 (16.2–30.7)	41.2 (37.0–46.6)^a^	30–45
Phenytoin	>40	15.2 (11.9–19.2)^a^	>40
Rufinamide	>40	>350^a^	<40
Tiagabine	6.2 (5.0–8.8)	8.0 (6.6–9.4)^a^	5–10
Topiramate	>300	299 (251–667)	~300
Valproate	126 (93–155)	351 (324–373)^a^	>300

Note

ED_50_ values were obtained using efficacy (# protected/N) values for each treatment group, as shown in Table 1.

a Metcalf et al 2017

b Upper confidence interval extended beyond the highest dose tested.

### 3.1 Sodium channel blockers

Several prototype sodium channel blockers were evaluated in lamotrigine‐resistant fully kindled rats (see Tables 1 and 2). Carbamazepine dose‐dependently reduced convulsive seizures (ED_50_ 42.1 mg/kg). Behavioral impairments were observed for carbamazepine at the highest doses tested (80 mg/kg). Although afterdischarge duration was reduced at two doses of carbamazepine (30 and 80 mg/kg) and one dose of lacosamide (30 mg/kg), these changes were not dose‐dependent. Eslicarbazepine, lacosamide, lamotrigine, phenytoin, and rufinamide had no little or no effect on seizure severity at the doses tested. Further, the maximum dose tested for each compound produces behavioral impairments, and therefore, no higher doses were attempted. Thus, with the exception of carbamazepine, seizures in this model were resistant to sodium channel blockers.

### 3.2 Drugs acting on GABA Receptors or Uptake

ASDs that potentiate the effects of GABA or increase GABA levels (reuptake inhibition) were also evaluated in fully kindled lamotrigine‐resistant rats. Clobazam dose‐dependently reduced seizures at the doses tested (5‐15 mg/kg; ED_50_ 9.2 mg/kg). However, afterdischarge duration was significantly increased at a 5 mg/kg dose of clobazam, and the cause for this change was not apparent. A dose of 15 mg/kg of clobazam was associated with behavioral impairments in all animals tested. Clonazepam also dose‐dependently reduced seizures (0.25‐4 mg/kg, ED_50_ 0.9 mg/kg), and afterdischarge duration was reduced at the highest doses tested (2‐4 mg/kg). The highest dose (4 mg/kg) also produced behavioral impairments in a majority of animals tested. Phenobarbital demonstrated dose‐dependent efficacy against convulsive seizures (15‐45 mg/kg, ED_50_ 23.3 mg/kg) and reduced afterdischarge duration at 30 and 45 mg/kg. In addition, 45 mg/kg produced behavioral impairments. Tiagabine dose‐dependently reduced seizures (2.5‐10 mg/kg, ED_50_ 6.2 mg/kg). Although afterdischarge duration was reduced at the highest dose tested (10 mg/kg), this dose was also associated with behavioral impairments in all animals tested. Data for GABA‐acting drugs are summarized in Tables 1 and 2. In summary, GABA‐acting drugs were generally effective in reducing or blocking seizures in lamotrigine‐resistant kindled rats.

### 3.3 Drugs acting on Ca^2+^ channels

Both ethosuximide (mechanism: T‐type Ca^2+^ channel modulation) and gabapentin (mechanism: binding of the α2δ subunit of voltage‐gated Ca^2+^ channels) were evaluated in this model (see Tables 1 and 2). Ethosuximide was evaluated at one dose (200 mg/kg), which did not reduce convulsive seizures (2/8 protected) or afterdischarge duration and was associated with behavioral impairments in all animals tested. By contrast, gabapentin dose‐dependently reduced convulsive seizures (10‐300 mg/kg; ED_50_ 268 mg/kg), and afterdischarge duration was reduced at the highest dose tested. All of the doses tested of gabapentin were well‐tolerated (see Tables 1 and 2). Therefore, efficacy for agents acting on Ca^2+^ channels was mechanism‐specific, as only gabapentin was effective in this model.

### 3.4 Drugs with Unique or Mixed Mechanisms of Action

ASDs with other unique or mixed mechanisms of action were also evaluated. Ezogabine (mechanism: modulation of K^+^ channels, M current) dose‐dependently reduced convulsive seizures and afterdischarge duration (1‐23 mg/kg; ED_50_ 3.2 mg/kg). However, the highest dose tested also produced behavioral impairment in all animals tested. Levetiracetam (mechanism: SV2A modulation) produced mild‐moderate convulsive seizure protection (50‐400 mg/kg), did not affect afterdischarge duration, and was well‐tolerated at all doses tested. Topiramate (mixed mechanism) was evaluated at a dose of 300 mg/kg, which did not reduce convulsive seizures or afterdischarge duration and was associated with behavioral impairments in a majority of animals tested. By contrast, valproate (mixed mechanism) dose‐dependently reduced seizures (75‐300 mg/kg, ED_50_ 126 mg/kg) and reduced afterdischarge duration at doses above 75 mg/kg. Further, valproate was well‐tolerated at all doses tested (see Tables 1 and 2). Therefore, ezogabine and valproate were effective in this model, while levetiracetam was only moderately efficacious and topiramate did not reduce seizures.

In order to confirm that reuse of fully kindled and lamotrigine‐resistant rats did not affect drug sensitivity, a fully efficacious dose of valproate was administered as the first and last compound for a cohort of rats tested a total of eleven times. Valproate retained full efficacy (8/8 protected, 7/7 protected) at doses of 300 mg/kg both times it was tested (data not shown).

## 4 DISCUSSION

The prevalence of drug‐resistant epilepsy warrants the use of models that demonstrate chronic susceptibility to seizures and neuropathological changes that occur in temporal lobe epilepsy.^13^ Lamotrigine and other ASDs, administered during various forms of kindling, have been used to induce pharmacoresistant seizures.^13^, ^27^, ^28^, ^29^, ^30^ More recently, this paradigm has been applied to the corneal‐kindled mouse, wherein lamotrigine is administered during the kindling process in order to develop lamotrigine‐resistant kindled mice^31^. The lamotrigine‐resistant amygdala‐kindled rat has become an important and useful model for the evaluation of novel compounds with potential activity against pharmacoresistant seizures. Despite the importance and prevalence of this model in preclinical evaluation of novel compounds,^13^ and although there have been previously published reports for the evaluation of some prototype compounds,^10^, ^11^ this is the first comprehensive evaluation of this model comparing prototype antiseizure compounds. In addition to presenting behavioral seizure and afterdischarge duration data on ASD prototypes, this report also describes noteworthy differences in tolerability between drug‐ and seizure‐experienced animals (ie, lamotrigine‐resistant, fully kindled) and TD_50_ values obtained in young adult, seizure‐ and drug‐naïve rats. This information may therefore inform the development of ASDs wherein late‐stage tolerability assessments should be performed in animals that sufficiently model the epilepsy disease state (eg, fully kindled rats or post‐status epilepticus‐induced spontaneously seizing rats).

The sodium channel blockers (carbamazepine, eslicarbazepine, lacosamide, lamotrigine, phenytoin, and rufinamide) were generally not efficacious in this model and were associated with untoward effects at the highest doses tested. With the exception of carbamazepine, which was moderately effective (protective index ~1.4), ED_50_ values could not be obtained for this class of drug. For carbamazepine, whose potency is >10‐fold lower than that observed in the maximal electroshock model,^32^ the highest dose tested was also associated with poor tolerability. Carbamazepine was previously shown to lose efficacy in this model, albeit at higher doses than observed in the present study.^10^ Taken together, these data are in agreement with previously reported observations wherein lamotrigine resistance extends to other sodium channel blockers.^2^, ^3^, ^10^ Cross‐tolerance to only compounds with similar mechanisms of action may represent a limitation of this model, as it may not adequately represent the clinical spectrum of pharmacoresistance. It is noteworthy, however, that cross‐tolerance may occur for distinct mechanistic classes, as it was observed that tolerance to carbamazepine in the amygdala kindling model can result from treatment with levetiracetam.^33^ In similar models, resistance may extend to compounds with other mechanisms of action such as retigabine and valproate, as seen in the lamotrigine‐resistant kindled mouse.^31^ It may also be the case that some sodium channel blockers are more likely to induce resistance, as it has been observed in phenobarbital‐resistant kindled rats that are also resistant to phenytoin.^5^, ^6^ Despite these observations, the mechanisms contributing to drug resistance in epilepsy, including the phenomenon of cross‐tolerance, are not fully understood.

When tolerability of ASDs was assessed in this model, lamotrigine showed a tolerability profile that is similar to that observed in young adult rats. Conversely, carbamazepine, lacosamide, and phenytoin were better tolerated in lamotrigine‐resistant fully kindled rats, as their estimated TD_50_ values in these animals are 1.5‐ to 3‐fold higher than those obtained in young adult rats. Interestingly, rufinamide and eslicarbazepine showed dramatic reductions in tolerability when compared to young, naive rats. The mechanisms contributing to these reductions in tolerability are not known at this time and were beyond the scope of this study. Understanding the reasons why seizures become refractory to sodium channel blockers may therefore provide insights into the underlying mechanism contributing to pharmacoresistance in patients.

Compounds targeting either GABA receptors or GABA‐uptake proteins produced dose‐dependent efficacy against convulsive seizures, and ED_50_ values were generally near or below estimated TD_50_ values obtained from minimal motor impairment assays. This is similar to previous studies which have demonstrated the efficacy of compounds acting on GABA in amygdala and hippocampal kindling models. For instance, clobazam, clonazepam, and phenobarbital all reduced seizures in amygdala kindling at comparable doses to those used in this study.^34^, ^35^, ^36^, ^37^ Similarly, tiagabine reduces seizures in amygdala kindling^38^ and hippocampal kindling at comparable doses.^39^ Further, GABA‐acting compounds were generally equally well‐tolerated, or better tolerated, in lamotrigine‐resistant kindled rats than in naïve rats. Therefore, this model appears to be sensitive to GABA‐acting compounds and resistant to sodium channel blockers.

Compounds acting to modulate Ca^2+^ channels show differential activity, depending on the specific mechanism. For example, ethosuximide was both poorly tolerated and ineffective at the dose tested (200 mg/kg), whereas it has previously demonstrated efficacy against amygdala‐kindled seizures.^36^ The lack of activity in lamotrigine‐resistant kindled rats is consistent with the clinical use of ethosuximide for generalized absence seizures, but not focal or generalized tonic‐clonic seizures.^40^ Conversely, gabapentin was moderately effective in this model, which is similar to previous work where gabapentin reduced seizure behavior in amygdala kindling.^9^ However, it is noteworthy that while gabapentin may be useful in partial‐onset seizures, it is perceived to have weak efficacy and is associated with notable side effects^41^, ^42^ in clinical populations. Interestingly, gabapentin was well‐tolerated in these studies, but it is noteworthy that tolerability observations in these studies were limited to motor impairment. Therefore, conclusions drawn from tolerability comparisons made in this study may not extend to all compounds. A more comprehensive behavioral battery assessment may be useful in order to better detect untoward effects of gabapentin in this model.

Ezogabine was more potent in this model than in the rat 6‐Hz model and was comparably effective in the maximal electroshock model.^19^ Ezogabine was previously shown to be effective in this model, albeit at a higher dose.^11^ Further, it appears that ezogabine is better tolerated in younger adult rats rather than kindled rats. Of note, however, this estimation does not effectively encapsulate the bladder retention and skin discoloration that have limited ezogabine's clinical utility.^40^ Valproate was also effective in this model at doses comparable to those effective in the maximal electroshock model and lower than those effective in the rat 6‐Hz model.^19^ Further, valproate has previously demonstrated efficacy against the amygdala‐kindled seizure model.^34^, ^36^, ^43^ Topiramate was not highly effective in this model and poorly tolerated in a majority of animals at the dose tested. Therefore, a lower dose of topiramate was not evaluated. These results contrast with those obtained in post‐kainate spontaneously seizing rats^44^ and in amygdala‐kindled rats,^45^ where topiramate reduced the incidence of seizures. This difference in efficacy may point to the pharmacoresistance of this model related to repeated drug exposure during the kindling process, though the two models differ in seizure etiology. It is also noteworthy that the TD_50_ obtained for topiramate in young adult naïve rats is greater than that reported previously.^19^ Interestingly, there have been previous discrepancies in published reports of the tolerability of topiramate.^46^, ^47^ Levetiracetam was ineffective in this model at doses up to 400 mg/kg. Levetiracetam reduces kindled seizures, despite not demonstrating efficacy in traditional screening models (maximal electroshock and pentylenetetrazol‐induced seizures).^48^ By comparison, levetiracetam was not able to fully reduce seizures in a model of hippocampal kindling.^39^ Further, levetiracetam was well‐tolerated in this model, which is consistent with a preclinical profile of a wide safety margin for this drug.^48^ In summary, for drugs with unique and mixed mechanisms of action, ezogabine and valproate were effective at well‐tolerated doses, whereas topiramate was not well‐tolerated and levetiracetam was ineffective at the doses tested.

A limitation of the current study was that each ASD was evaluated only after a single (acute) administration. Therefore, it is not clear whether the effects described herein would continue or change with repeated administration. While this model is useful for differentiation of potentially novel ASDs,^12^ it should be noted that without repeated administration and testing it is difficult to interpret how these data will translate into clinical pharmacoresistant populations. Another limitation of the present study is that pharmacological profiles for amygdala‐kindled rats not treated with lamotrigine were not obtained. While this was beyond the scope of the present study, comparisons are made (when possible) to previous studies evaluating ASDs in amygdala kindling.

Drug‐resistant epilepsy is defined as a lack of response to two or more ASDs. Further, resistance in patients is not due to treatment with any particular drug or drug class.^49^ It is therefore worth noting that screening and evaluation of ASDs or novel therapies using lamotrigine tolerance as a predictor of drug resistance across the epilepsy population may be useful, but does not fully encapsulate all potential causes of resistance. We observed that in addition to sodium channel blockers, compounds acting through other mechanisms were also ineffective in this model as compared to amygdala kindling alone. Levetiracetam did not reduce seizure scores at a dose more than ten times greater than that demonstrated to reduce seizures in amygdala‐kindled rats.^48^ Similarly, gabapentin showed only moderate reductions in seizure scores at doses up to 300 mg/kg where lower doses are effective in amygdala‐kindled rats.^50^, ^51^ This suggests that, as in human epilepsy populations, drug tolerance in this model occurs for multiple ASDs. However, drug resistance arises not only from pharmacodynamic tolerance, but also from other mechanisms.^52^ These include pharmacokinetic mechanisms (changes in transporter expression or hepatic metabolism), network changes that reduce the likelihood of drug responsiveness, and gene expression changes.^53^ The extent to which these additional mechanisms may have contributed to the lack of or reduced effects of compounds in this model was beyond the scope of the present study. Nevertheless, these complementary mechanisms will be an important component of future use of this model.

In summary, there were few compounds that were highly effective in this model, as defined by ED_50_ values at least 2‐fold lower than the approximate TD_50_ in lamotrigine‐kindled rats. These compounds include carbamazepine, clonazepam, ezogabine, and valproate, which comprise distinct mechanistic classes. In addition, clobazam, ezogabine, phenobarbital, tiagabine, and valproate all have ED_50_ values that are lower than the TD_50_ values obtained in young adult, seizure‐naïve rats. Of these, only ezogabine and valproate fit the criteria of having ED_50_ values at least 2‐fold lower than the TD_50_ collected in younger rats. This suggests that therapeutic indices (ie, TD_50_/ED_50_) derived from seizure‐experienced and/or drug‐experienced rats may not be comparable for many drugs. This finding may have larger implications, as therapeutic index can be a key deciding factor in whether or not a preclinical drug candidate advances through drug development pipelines. While TD_50_ determinations in kindled rats were not performed in this study, future studies should include these determinations in order to better estimate therapeutic indices in seizure‐experienced rats.

## CONFLICT OF INTEREST

None of the authors has any conflict of interest to disclose. We confirm that we have read the Journal's position on issues involved in ethical publication and affirm that this report is consistent with those guidelines.
